# Low sample volume origami-paper-based graphene-modified aptasensors for label-free electrochemical detection of cancer biomarker-EGFR

**DOI:** 10.1038/s41378-020-0146-2

**Published:** 2020-05-18

**Authors:** Yang Wang, Shuai Sun, Jinping Luo, Ying Xiong, Tao Ming, Juntao Liu, Yuanyuan Ma, Shi Yan, Yue Yang, Zhugen Yang, Julien Reboud, Huabing Yin, Jonathan M. Cooper, Xinxia Cai

**Affiliations:** 1 State Key Laboratory of Transducer Technology, Institute of Electronics, Chinese Academy of Sciences, Beijing, 100190 China; 2 University of Chinese Academy of Sciences, Beijing, 100190 China; 3 Key laboratory of Carcinogenesis and Translational Research (Ministry of Education), Department of Thoracic Surgery II, Peking University Cancer Hospital & Institute, Beijing, 100142 China; 4 Division of Biomedical Engineering, James Watt School of Engineering, University of Glasgow, Oakfield Avenue, Glasgow, G12 8LT United Kingdom

**Keywords:** Biosensors, Microfluidics, Nanoparticles, Nanoparticles

## Abstract

In this work, an electrochemical paper-based aptasensor was fabricated for label-free and ultrasensitive detection of epidermal growth factor receptor (EGFR) by employing anti-EGFR aptamers as the bio-recognition element. The device used the concept of paper-folding, or origami, to serve as a valve between sample introduction and detection, so reducing sampling volumes and improving operation convenience. Amino-functionalized graphene (NH_2_-GO)/thionine (THI)/gold particle (AuNP) nanocomposites were used to modify the working electrode not only to generate the electrochemical signals, but also to provide an environment conducive to aptamer immobilization. Electrochemical characterization revealed that the formation of an insulating aptamer–antigen immunocomplex would hinder electron transfer from the sample medium to the working electrode, thus resulting in a lower signal. The experimental results showed that the proposed aptasensor exhibited a linear range from 0.05 to 200 ngmL^−1^ (*R*^2^ = 0.989) and a detection limit of 5 pgmL^−1^ for EGFR. The analytical reliability of the proposed paper-based aptasensor was further investigated by analyzing serum samples, showing good agreement with the gold-standard enzyme-linked immunosorbent assay.

## Electrochemistry: Ultrasensitive detection of cancer biomarker using graphene-modified biosensors

A low-cost electrochemical paper-based sensor has been developed for the highly sensitive detection of epidermal growth factor receptor (EGFR). EGFR normally activates relevant genes, resulting in cell division and proliferation, although its overexpression has been observed in some forms of cancer. There is thus a growing interest in determining EGFR levels as a biomarker for early-stage disease diagnosis. A team headed by Xinxia Cai at the Chinese Academy of Sciences, Beijing succeeded in developing a biosensor for EGFR by combining the screen printing of a graphene-modified nanocomposite electrode onto an origami paper-folding for enabling microfluidic reagent and sample processing. The device gave a low detection limit and linear responses across a wide range of EGFR concentrations in human serum (electronic input values). The authors believe that their device can be easily adapted to other soluble biomarkers and that it has potential for use as a handheld instrument as point-of-care testing in early diagnosis and efficacy evaluation of cancer.

## Introduction

Epidermal growth factor receptor (EGFR), with tyrosine kinase activity, is a transmembrane glycoprotein^1,2^. Once bound to its specific ligands, it can activate specific genes, promoting cell division and proliferation^3^. The normal EGFR concentration in humans is in the range of 1–25 ngml^−1^ (ref. ^4^); however, overexpression of EGFR occurs in a variety of carcinomas, including gastric, breast, ovarian, and colorectal cancers^5^. For example, the concentration of EGFR in the lymph node metastasis of lung cancer patients can be as high as 850 ngml^−1^ (ref. ^6^). Hence, there has been increasing interest in the determination of EGFR levels as a basis for early-stage cancer identification^7^.

In clinical laboratories, various technologies, including enzyme-linked immunosorbent assay (ELISA)^8^, western blotting^9^, and immunohistochemistry (IHC)^10^, have been developed for the detection of EGFR. For example, Atkins et al. used an IHC-based screening method for the detection of EGFR with the EGFR pharmDX kit^11^. However, these techniques face challenges ranging from their detection ranges, sensitivities, assay complexity to the cost of equipment^12^^,13^. To overcome these limitations, electrochemical detection methods have been developed for the quantitative detection of EGFR. For example, Ilkhani et al. reported a new electrochemical aptamer/antibody-based sandwich immunosensor with a linear sensing range of 1–40 ngmL^−1^ and a detection limit of 50 pgmL^−1^ (ref. ^5^). Although these methods are relatively simple, easy to perform, and can be used in point-of-care testing (POCT) formats, they have limited effectiveness. Thus, efforts are underway to develop label-free quantitative assays with low system complexity for monitoring the concentrations of EGFR in real time. Label-free electrochemical assays, which can directly convert a biological recognition event, such as ligand binding of a biomarker, into a measurable electrical signal, have the potential to address many of the abovementioned issues^14,15^.

Microfluidic devices have been widely used as analysis platforms, as a consequence of the need for convenient POCT formats^16,17^. Currently, most microfluidic analytical devices are fabricated using glass, silicon, or polymeric channels, the latter involving soft-lithography of polydimethylsiloxane^18^ or polymethyl methacrylate^19^, for example. However, these devices usually require complex fabrication processes and external pumping forces for liquid transportation, which largely restrict their applications. Thus, microfluidic paper-based devices (μPADs)^20^, which are fabricated on paper, have gained considerable attention as alternative platforms for detection. They are attractive because of advantages, such as their relatively low cost. The paper matrix enables flow through capillary forces, which facilitates the transportation of samples and reagents without the need for external equipment^21^. Moreover, many low-cost methods have been demonstrated for μPADs fabrication, including photolithography^22^, ink jet printing^23^, plasma treatment^24^, laser treatment^25^, screen printing^26^, and wax printing^27^. Among all these methods, wax printing is a rapid and easy method for producing large-scale µPADs with no requirement for a clean room facility^28^. The cost for µPADs fabrication based on wax printing is also modest. Due to these advantages, wax-printed paper has become an attractive substrate in the field of detection^29^.

Currently, different detection mechanisms have been used in μPADs for point-of-care applications^30^. In 2007, the Whitesides research group at Harvard University first reported a novel μPAD to analyze glucose and proteins in urine by colorimetry^20^. The qualitative results of the assay could be read directly by the naked eye or semiquantitatively with digital equipment. In an effort to conduct quantitative analysis for diagnostic tests based on μPADs, electrochemical detection methods have been successfully introduced onto the device^31^—providing an attractive alternative detection scheme for paper-based microfluidics due to their high sensitivity and high selectivity^32^. An additional advantage of the electrochemical method is the simplicity of the instrumentation, resulting in low electrical power requirements for handheld or in-field use^33^.

Aptamers are DNA or RNA sequences that are selected in vitro by the mature technology systematic evolution of ligands by exponential enrichment^34,35^. Due to their ability to bind with specific molecular targets, they have been widely regarded as promising recognition elements for biosensor applications, particularly for the detection of protein analytes^36^. In addition to exhibiting high affinity, they also possess several key advantageous properties, including ease and cost of production, as well as their potential for chemical modification to yield improved and tailored properties, providing improved stability and shelf life at ambient temperature, compared with antibodies;^37–39^ lack of aggregation; as well as good pH and temperature tolerance—all making the proposed approach potentially suitable for future clinical applications^40^. Combining the attractive properties of aptamers with the distinct advantages of electrochemical detection methods has received increasing attention. Electrochemistry offers innovative routes for interfacing aptamer interactions with the signal-generating element and for obtaining protein diagnostics in a simple, fast, and inexpensive manner^41^. To enhance the sensitivity and selectivity of electrochemical aptasensors, a variety of nanomaterials have been employed to modify electrodes^42^. For example, graphene has distinct electrical properties^43^ and graphene-modified electrodes not only facilitate the transfer rate of electrons, but also provide a large surface for biomolecule immobilization, resulting in improved sensitivity^44^.

Here, we demonstrated an origami-paper-based device employing an electrochemical detection method for highly sensitive and POCT of EGFR. The hydrophobic and hydrophilic areas on the device, to define fluid channels were created by the printing of wax, while electrochemical electrodes were made by screen printing. The device enabled sample processing and detection by simply folding the paper to enable flow or valving. Amino-functionalized graphene (NH_2_-GO)/thionine (THI)/gold particle (AuNP) nanocomposites were synthesized to modify the working electrode (WE) not only to generate a response, but also to help immobilize aptamers through Au-S bonds. The anti-EGFR aptamers were presynthesized and modified with thiol groups. A simple electrochemical detection method that avoided labeling either antigen or aptamer was adopted. The principle of the biosensor was based upon the fact that the electron transfer rate would be inhibited with the formation of aptamer–antigen bioconjugates, resulting in a decrease in the measured current. The origami-paper-based device, with low cost and low sample consumption, offers an alternative, promising platform for EGFR detection for POCT sensing in early diagnosis and efficacy evaluation of cancer.

## Materials and methods

### Reagents and apparatus

The anti-EGFR aptamer, modified with thiol groups on its 5′-end, was purchased from Sangon Biotechnology Co. Ltd. (Shanghai, China). The aptamer was purified by high-performance liquid chromatography, and its sequence was 5′-TAC CAG TGC GAT GCT CAG TGC CGT TTC TTC TCT TTC GCT TTT TTT GCT TTT GAG CAT GCT GAC GCA TTC GGT TGA C-3′ (Mw 23474.2 gmol^−1^). Whatman No. 1 chromatography paper was purchased from GE Healthcare worldwide. The inks, including conductive carbon ink (ED581ss), were purchased from Acheson, and Ag|AgCl ink (CNC-01) was purchased from Yingman Nanotechnology Company (China). EGFR antigens, EGFR ELISA kits, and tris-ethylenediaminetetraacetic (TE) buffer solution (pH = 8.0) were also purchased from Sangon Biotechnology Co. Ltd. Thionin acetate was obtained from Alfa Asear, and NH_2_-GO was purchased from Nanjing Xianfeng Nanomaterials Company (China). All reagents were of analytical grade and used without further purification.

Electrochemical characterization and analytical measurements, including cyclic voltammetry (CV) and differential pulse voltammetry (DPV), were carried out on an Autolab PGSTAT302N electrochemical workstation (Autolab, Herisau, Switzerland). A Xerox ColorQube 8570 digital wax printer was used to fabricate hydrophobic areas on paper. Ultrapure water was purified by a Michem water apparatus (Michem, China, resistivity > 18 MΩ). The morphology of the synthesized nanocomposites was characterized by a Hitachi H7650 transmission electron microscope (Hitachi, Japan).

### Design and fabrication of origami-paper-based device

**Fig. 1:**
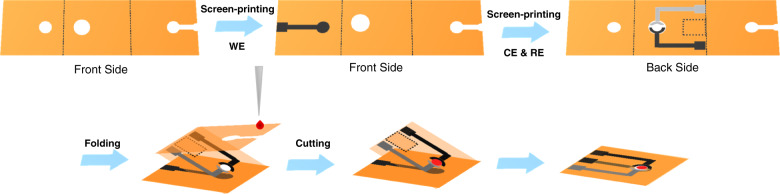
Design and fabrication process of the origami-paper-based device

A simple illustration of the origami-paper-based device, which was designed using Adobe Illustrator CS5, is shown in Fig. 1. The devices were directly fabricated by a wax printer without needing any cleanroom access. Wax was printed onto the surface of chromatography paper to selectively create hydrophobic area (colored) and hydrophilic channels (white). The wax-patterned filter paper was then heated at 120 °C for 5 min in an oven, letting the wax melt and penetrate into the paper in the *z*-direction, creating a hydrophobic barrier through the whole thickness of the paper (*z*) rather than just on the surface. Subsequently, three electrodes were screen printed onto the device. The WE was fabricated on the front of the device using conductive carbon ink. The carbon ink counter electrode and Ag|AgCl ink reference electrode (RE) were screen printed on the back side.

### Preparation of origami-paper-based EGFR aptasensor

**Fig. 2:**
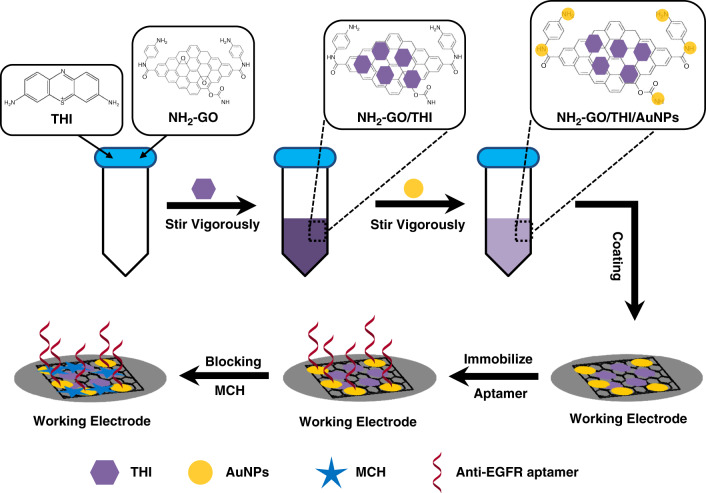
Modification process of the origami-paper-based EGFR aptasensor

A schematic illustration of the origami-paper-based EGFR aptasensor preparation process is shown in Fig. 2. Briefly, NH_2_-GO/THI/AuNP nanocomposites were first synthesized and then coated onto the surface of the WE to help immobilize anti-EGFR aptamers. The specific modification process can be described as follows.

For the preparation of NH_2_-GO/THI/AuNP nanocomposites, 2.0 mg of NH_2_-GO was first dispersed into 2.0 mL of ultrapure water by ultrasonication for half an hour, followed by the addition of 2.0 mL of stable THI solution (2.0 mgmL^−1^). The above mixture was stirred vigorously for 24 h at room temperature to obtain NH_2_-GO/THI nanocomposites due to the π–π stacking interactions between THI and graphene. A total of 1.0 mL of AuNP solution was mixed with 200.0 μL of the as-prepared NH_2_-GO/THI nanocomposites and stirred for another 12 h. The AuNP solution was presynthesized with diameters of ~15 nm. Eventually, NH_2_-GO/THI/AuNP nanocomposites were obtained through several centrifugation and washing steps.

As shown in Fig. 2, 10.0 μL of synthesized NH_2_-GO/THI/AuNP nanocomposites were coated on the WE surface and dried at 50 °C in an oven. A total of 10.0 μL of 1 mM anti-EGFR aptamers were covalently immobilized on the modified WE through Au-S bonds. Subsequently, 10.0 μL of mercaptoethanol solution, whose concentration was 1.0 mM, was used to block the nonspecific binding sites at room temperature for 1 h. Finally, TE buffer (10.0 mM Tris-HCl and 1.0 mM EDTA) was employed to wash the fabricated origami-paper-based aptasensor several times. The device was stored at 4 °C.

### Electrochemical measurements of EGFR

The performance of the abovementioned origami-paper-based aptasensor was evaluated by assaying standard solutions of EGFR. 5.0 μL of biological sample (e.g., serum), spiked with different EGFR concentrations in the range between 1.0 pgmL^−1^ and 500 ngmL^−1^, were introduced from the sample inlet hole located at the side of the device. The devices were stored for up to 1 h before electrochemical measurement. A solution of 0.1 M PBS solution (pH = 7.4) was added to the sample and an Autolab electrochemical workstation was used to perform the electrochemical characterization. CV and DPV were utilized for the characterization of the fabricated paper-based aptasensor because they were accurate and highly sensitive. The typical CV responses were recorded at a scan rate of 100 mVs^−1^ between −0.5 V and 0.1 V. In addition, the DPV responses of various concentrations of EGFR were obtained under conditions of 5.0 mV step potential, 0.025 s modulation time, and 0.5 s interval time in the range of −0.5 V to 0.1 V.

## Results and discussion

### Working principle of the origami-paper-based electrochemical aptasensor

The origami-paper-based device was separated into three sheets. Three electrodes were screen printed onto different sheets. Once a sample was added from the sample inlet, it would flow through the microchannel and permeate to the surface of the WE to enable electrochemical detection. The WE in this device was designed to be separated from the counter, and REs to reduce sample consumption and prevent the contamination of auxiliary electrodes during the modification process. Compared with devices that require complex networks of channels, the origami device fabricated in this work would ensure a more rapid test, as the time taken for fluid to permeate was shortened. Moreover, the exclusion of some small channels would lead to a low sample consumption of ~4.7 μL and an increase in sensitivity. Finally, the device was easy to insert into the backend interfaces so that the detection results could be directly read by the electrochemical workstation.

### Characterization of synthesized NH_2_-GO/THI/AuNP

**Fig. 3:**
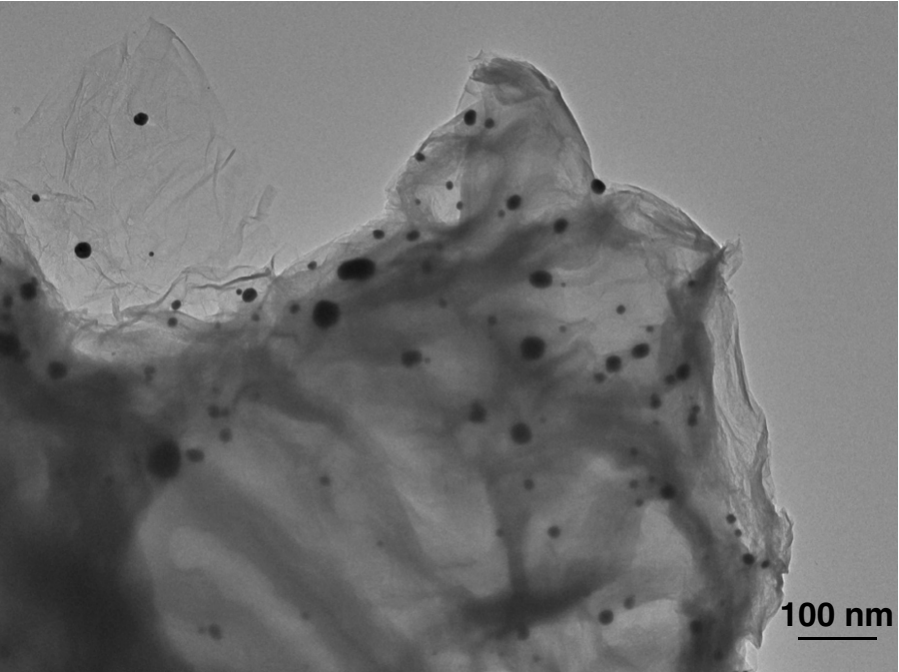
TEM image of the synthesized NH_2_-GO/THI/AuNP nanocomposites

**Fig. 4:**
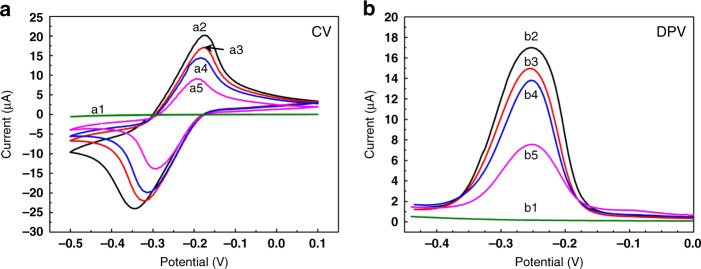
Electrochemical properties of the origami-paper-based EGFR aptasensor monitored in 0.1 M PBS solution (pH = 7.4). **a** CV responses of the stepwise preparation processes of the aptasensor: (a1) bare working electrode (WE); (a2) NH_2_-GO/THI/AuNP nanocomposite-modified WE; (a3) WE with immobilized EGFR aptamers; (a4) after incubation with 5 pgmL^−1^ EGFR solution and with (a5) 1 ngmL^−1^ EGFR solution; **b** DPV responses of stepwise fabrication processes of the aptasensor following the same order (b1–b5) as in **a**

NH_2_-GO/THI/AuNP nanocomposites were synthesized to enhance the electrochemical properties of the screen-printed WE. They also enabled the immobilization of anti-EGFR aptamers by using the self-assembly interactions between Au and thiol groups on the aptamer. To characterize the synthesized nanocomposites, transmission electron microscopy (TEM) images were obtained. As shown in Fig. 3, NH_2_-GO showed corrugated structures, which effectively increased the surface area of the electrode. THI, whose molecular formula is displayed in Fig. 2, noncovalently attached to the NH_2_-GO surface because of the π–π stacking interactions between benzene rings. A larger surface area meant that more THI molecules could be loaded onto the electrode surface. As electroactive materials, THI molecules generate currents through redox reactions. As a result, more THI molecules present led to a higher electrochemical response. In addition, the TEM image also demonstrates that a large number of AuNPs were distributed on the surface of NH_2_-GO, and no significant agglomeration was observed. The diameters of the AuNPs ranged from 10 nm to 50 nm. AuNP has unique properties due to the quantum size effect. They have a strong affinity for electrons, thus acting as strong electron acceptors^45–47^, leading to an increase in the anodic peak current (Fig. 4). From the image, we could conclude that the nanocomposites were synthesized successfully.

### Electrochemical properties of the EGFR aptasensor

The fabrication process of the origami-paper-based EGFR aptasensor and its corresponding electrochemical behavior were characterized with both CV and DPV methods in a 0.1 M PBS solution (pH = 7.4). The typical responses of CV as a result of stepwise preparation processes are shown in Fig. 4a. The bare WE (curve a1) showed no oxidation or reduction behavior. In comparison, a well-behaved electrochemical response was observed under the same conditions after modification with NH_2_-GO/THI/AuNP nanocomposites (curve a2), indicating that a conformal coating of the nanocomposites was formed on the WE. Two pronounced redox peaks, which were attributed to the THI molecules, were observed at −0.18 V and −0.31 V.

The magnitude of the electrochemical response was lower than that of the nanocomposite-modified electrodes when EGFR aptamers were immobilized onto the electrode surface (curve a3). It appears that the presence of insulating aptamers hindered the process of electron transfer, resulting in a decrease in the current response. Subsequently, when different concentrations of EGFR antigens were added (curve a4 and curve a5), the current intensity decreased with increasing concentrations, confirming the fact that once the EGFR antigen bound to the aptamer, they would influence electron transfer from the medium to the WE. Furthermore, more insulating immunocomplexes would form with a higher concentration of antigens, which would, in turn, result in a lower electrochemical current. The CV results indicated the successful fabrication and function of the electrochemical paper-based aptasensor.

Similar behaviors were observed when DPV was utilized for the characterization of the aptasensor. The results obtained from DPV studies were correlated with those from CV carried out under similar conditions. As shown in Fig. 4b, compared with the bare WE (curve b1), the NH_2_-GO/THI/AuNP-modified WE exhibited a pronounced DPV response (curve b2), with a peak current of 16.99 μA. Then, the current response dropped to 14.98 μA upon immobilization of aptamers (curve b3). Moreover, with the addition of different concentrations of EGFR antigen (curve b4 and curve b5), the magnitude of the DPV response was observed to decrease gradually. The CV and DPV results were highly consistent with the principle of the electrochemical method mentioned earlier, which indicated that the aptasensor was modified successfully and enabled the detection of EGFR.

### Optimization of the experimental conditions for the EGFR aptasensor

To obtain a high and stable electrochemical response, factors that influence the electrochemical currents were optimized. During the electrochemical measurements, the electrochemical currents were derived from the THI molecules. Different concentrations of THI molecules led to different electrochemical currents. AuNP provided a suitable microenvironment for the immobilization of anti-EGFR aptamers and enhanced charge transfer. It was necessary to study the effect of the number of AuNPs. In addition, instead of antibodies, aptamers were used as the recognition element in this work. Therefore, the incubation time would also affect the performance of the electrochemical aptasensor. The effects of the three abovementioned conditions were investigated.

**Fig. 5:**
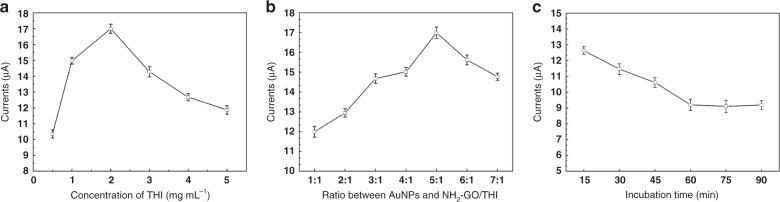
Optimization of parameters affecting DPV signals in the origami-paper-based aptasensor. **a** Effect of different concentrations of THI solutions on the amplitude of the peak current of the amperometric response of the device; **b** effect of different ratios of AuNPs on the DPV response of the device; **c** effect of the incubation time on the DPV response to 1 ngmL^−1^ EGFR

To choose an optimal concentration of THI molecules for the following electrochemical detection, a series of THI solutions were prepared with concentrations ranging between 0.5 mgmL^−1^ and 5.0 mgmL^−1^ to study its effect on the response. Different concentrations of THI solutions were mixed with a 1.0 mgmL^−1^ NH_2_-GO solution at a ratio of 1:1 to obtain NH_2_-GO/THI nanocomposites and their responses are reported in Fig. 5a. It can be observed from the figure that the DPV response reached a maximum at 2.0 mgmL^−1^.

The AuNP played an important role in the electrochemical performance of the aptasensor. Different amounts of AuNPs were mixed with the synthesized NH_2_-GO/THI nanocomposites at ratios of 1:1, 2:1, 3:1, 4:1, 5:1, 6:1, and 7:1. As shown in Fig. 5b, the peak current of the electrochemical aptasensor increased with the AuNP amount until the ratio of 5:1 and then began to decrease with further increases, when their amount could screen the charge transport to the electrode.

Incubation time is another key factor that influences performance linked to the status of the EGFR binding. The modified aptasensor was incubated with 1.0 ngmL^−1^ EGFR for 15, 30, 45, 60, 75, and 90 min. As shown in Fig. 5c, the peak current decreased gradually with increasing incubation time and tended to level off after 60 min. As a result, 60 min was selected as the incubation time for the following experiments.

### Analytical performance of the EGFR aptasensor

**Fig. 6:**
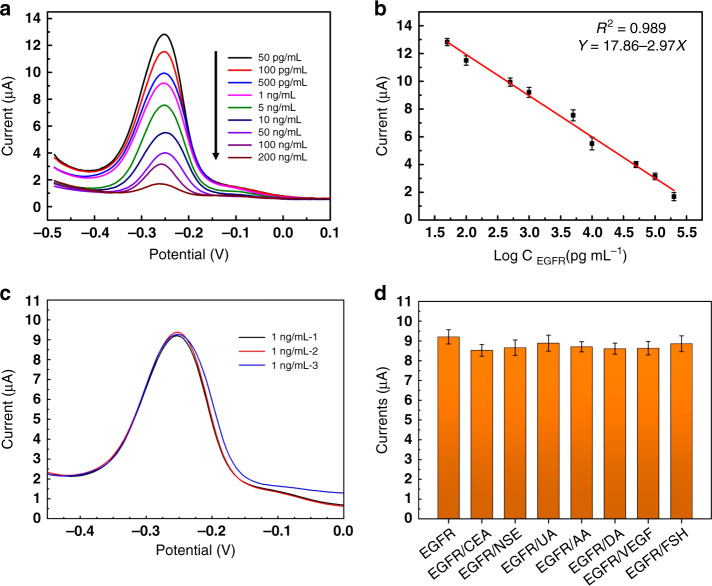
Assay results of standard EGFR solutions in 0.1 M PBS (pH = 7.4). **a** DPV responses of the origami-paper-based aptasensor to different concentrations of standard EGFR solutions ranging from 0.05 ngmL^−1^ to 200 ngmL^−1^; **b** peak current of DPV response as a function of the concentration of EGFR; **c** three repeated measurements of 1 ngmL^−1^ of EGFR; **d** selectivity of the aptasensor to 1 ngmL^−1^ of EGFR, and 1 ngmL^−1^ of EGFR mixed with 10 ngmL^−1^ solutions of some potentially interfering substances, carcinoembryonic antigen (CEA), neuron-specific enolase (NSE), uric acid (UA), ascorbic acid (AA), dopamine (DA), vascular endothelial growth factor (VEGF), and follicle-stimulating hormone (FSH). Error bars in **c** and **d** are standard deviations over three independent measurements

Under the optimized conditions described above, the electrochemical responses of the origami-paper-based aptasensor were recorded as a function of EGFR concentration. As shown in Fig. 6a, the electrochemical response of the aptasensor decreased continuously with increasing concentrations of EGFR antigen. Figure 6b shows that this decrease is linear (on a log scale, *R*^2^ = 0.989). The limit of detection (LOD) of the aptasensor can be concluded to be <5.0 pgmL^−1^, which was more sensitive than that of the commercial EGFR ELISA kit. The boosted electrochemical sensitivity of the aptasensor could be attributed not only to the nanocomposites, which increased the current response, but also to the key advantageous properties of aptamers over antibodies. Comparing the characteristics of the present study with other platforms (Table 1) indicated an excellent linear range and a low detection limit for the detection of EGFR, considering the ease-of-use and low cost of the platform with respect to the other methods mentioned.

**Table 1:** Comparisons of different kinds of biosensors and their analytical properties toward EGFR

No.	Method	Linear range	LOD	References
1	Impedimetric immunosensor modified with gold nanoparticles	1 pgmL^−1^ to 1 μg·mL^−1^	0.34 pgmL^−1^	^2^
2	Gold nanoparticles-modified capacitive sensor	20–1000 pgmL^−1^	20 pgmL^−1^	^49^
3	Antibody immobilized quartz crystal microbalance	0.01–10 μgmL^−1^	100 ngmL^−1^	^50^
4	Electrochemical aptamer/antibody-based sandwich immunosensor	1–40 ngmL^−1^	50 pgmL^−1^	^5^
5	DTSP-modified electrochemical immunosensor	0.01–100 ngmL^−1^	1 pgmL^−1^	^7^
6	Origami-paper-based electrochemical aptasensor	0.05–200 ngmL^−1^	5 pgmL^−1^	This work

### Repeatability and selectivity of the EGFR aptasensor

The repeatability of an electrochemical device, especially for such low-cost disposable paper-based format, is a key factor for practical applications. In this work, the repeatability was characterized by assaying 1 ngmL^−1^ EGFR antigen with three different devices that were fabricated in the same batch. As shown in Fig. 6c, the three independent aptasensors showed changes in current responses of 9.203 μA, 9.328 μA, and 9.270 μA. The coefficient of variation of the measurements was 0.79% (<5%), showing excellent repeatability.

Selective determination of target analytes is of significant analytical importance in clinical chemistry. To examine the selectivity of our aptasensor, potential interferents, carcinoembryonic antigen, neuron-specific enolase, uric acid, ascorbic acid, dopamine, vascular endothelial growth factor, and follicle-stimulating hormone were mixed with 1.0 ngmL^−1^ EGFR at a much larger concentration of 10.0 ngmL^−1^ for control experiments. As shown in Fig. 6d, no significant current change was observed. Although the concentration of interference was ten times higher than our detection target, the current variation was <7.42% of that without the potentially interfering species.

### Analytical results of serum samples

The practicality and analytical reliability of the paper-based aptasensor were further investigated by assaying five serum samples spiked with different concentrations of EGFR antigens ranging from 0.37 ngmL^−1^ to 4.35 ngmL^−1^, compared with measurements obtained by ELISA, which was used as a reference method (the ELISA kit enabled a detection range only of 0.1–8.0 ngmL^−1^). Table 2 shows no significant deviation between these two methods, ranging from 3.58 to 7.32%. In general, the range of EGFR in healthy individuals is ~1.0–25.0 ngmL^−1^. Moreover, the cutoff concentration to differentiate between a primary diagnostic and a diagnosis of metastasis is ~30.0 ngmL^−1^ (ref. ^48^). The detection results thus confirmed that our proposed EGFR aptasensor enabled sufficient sensitivity for clinical applications.

**Table 2:** Assay results of spiked serum samples using the EGFR aptasensor and ELISA kit

Serum samples	1	2	3	4	5
ELISA (ngmL^−1^)	4.35	3.44	1.27	0.82	0.37
Proposed aptasensor (ngmL^−1^)	4.56	3.25	1.22	0.88	0.40
Relative deviation (%)	4.72	−5.44	−3.58	6.96	7.32

## Conclusion

We have demonstrated an origami-paper-based label-free electrochemical aptasensor for ultrasensitive detection of EGFR. The device enabled a linear analytical response range across a wide range of concentrations and a low LOD. In summary, the proposed aptasensor combines low-cost paper-based microfluidic devices with a highly sensitive electrochemical detection. Adopting the concept of origami further simplified the device and reduced the sample consumption. The introduction of graphene-based nanocomposites onto the WE surface efficiently accelerated the electron transfer rate and further enhanced the detection signal. The aptamer used as the recognition element during detection enabled highly specific detection. The method provides several key advantages, including ease and low cost of production, as well as simple chemical modification to yield improved and tailored properties. The aptasensor described in this work could be easily adapted for other clinical or environmental applications. Moreover, many existing manufacturers have been able to integrate powerful electro-analysis functions into a handheld potentiostat. Once combined with these commercially available handheld instruments, the proposed paper-based aptasensor would have the potential in early diagnosis and efficacy evaluation of cancer.
